# Association between radiological findings and severity of community-acquired pneumonia in children

**DOI:** 10.1186/1824-7288-39-56

**Published:** 2013-09-13

**Authors:** Maria Francesca Patria, Benedetta Longhi, Mara Lelii, Carlotta Galeone, Maria Angela Pavesi, Susanna Esposito

**Affiliations:** 1 Pediatric Clinic 1, Department of Pathophysiology and Transplantation, University of Milan, Fondazione IRCCS Ca’ Granda Ospedale Maggiore Policlinico, 20122 Milan, Italy; 2 Luigi Devoto Department of Occupational Health, University of Milan, Milan, Italy; 3 Pediatric Radiology Unit, Fondazione IRCCS Ca’ Granda Ospedale Maggiore Policlinico, Milan, Italy

**Keywords:** CAP, Chest radiograph, Community-acquired pneumonia, Pneumonia

## Abstract

**Background:**

There are few published data concerning radiological findings and their relationship with community-acquired pneumonia (CAP) severity. The aim if this study was to assess radiographic findings in children with CAP of different severity in order to evaluate whether some parameters are associated with severe CAP.

**Methods:**

We analysed the characteristics of parenchymal densities in 335 chest radiographs of otherwise healthy children (173 males; mean age ± standard deviation, 7.5 ± 4.5 years) admitted to our Emergency Room for CAP. Upon admission, chest radiographs were obtained in the two standard projections, and the children with severe or mild/moderate CAP were compared in order to identify any correlations between CAP severity and the radiological findings.

**Results:**

Seventy-six of the 335 enrolled children (22.7%) fulfilled the criteria for severe CAP. In comparison with the children with mild/moderate CAP, in severe CAP there was a significantly greater frequency of a bilateral multifocal distribution (p = 0.01), the simultaneous involvement of ≥3 sites (p = 0.007), and the involvement of the right hilum (p = 0.02). The same results were confirmed in the multiple logistic regression model.

**Conclusions:**

This study shows that radiological findings such as a multifocal bilateral distribution, the simultaneous involvement of at least three sites, and right hilar consolidation are associated with severe CAP in otherwise healthy children, and could be considered markers of disease severity in children with CAP.

## Background

Community-acquired pneumonia (CAP) is a frequent childhood illness throughout the world, and a common reason for hospital admission [1,2]. The diagnosis of CAP in children is mainly based on clinical symptoms, and severe CAP is defined as the presence of fever, respiratory distress and/or dehydration [1,2]. There are differences between recently published guidelines in terms of the clinical criteria of severity, although all of the experts agree in considering hypoxemia the most important parameter for decision making [3-5]. Chest radiography is frequently performed in the Emergency Room even in mild to moderate cases, although appropriateness of performing chest radiography in children with CAP is still on debate and the actual guidelines recommend it only in order to verify the presence or absence of complications in patients with severe CAP [3-5]. There is evidence supporting the fact that radiographic findings did not correlate to any etiology [6], and chest radiography findings are not considered among the criteria for defining CAP severity. However, as there few published data concerning the relationship between the two, the aim of this study was to assess radiographic findings in children with CAP of different severity in order to evaluate whether some parameters are associated with severe CAP.

## Methods

We analysed the characteristics of parenchymal densities in 335 chest radiographs of otherwise healthy children (173 males; mean age ± standard deviation [SD], 7.5 ± 4.5 years), who were enrolled by the Respiratory Disease Section of Pediatric Clinic 1, University of Milan, Milan, Italy, between January 2009 and December 2010 after being admitted to the Emergency Room because of CAP. Otherwise healthy children aged <14 years with clinical signs such as tachypnea and abnormal breath sounds, and a chest radiograph consistent with CAP were considered eligible for the study. All of the chest radiographs were evaluated by an independent expert radiologist (MAP) who was unaware of the patients’ clinical and laboratory findings in accordance with the World Health Organisation (WHO) criteria for the standardised interpretation of pediatric chest radiographs for a diagnosis of pneumonia [7]. Patients suffering from severe chronic underlying diseases, such as cystic fibrosis, bronchodysplasia, primary ciliary dyskinesia, swallowing dysfunction, immunodeficiency, congenital heart defects, neurological diseases or malformations were excluded. Other 149 children (70 males; mean age ± SD, 2.1 ± 3.3 years) with a clinical diagnosis of CAP during the study period were not enrolled in the study because they were followed only in the Emergency Room and chest radiographies were not performed or were not available for evaluation in these patients. The study was approved by the Ethics Committee of Fondazione IRCCS Ca’ Granda, Ospedale Maggiore Policlinico, Milan, Italy, and written informed consent was obtained from the parents or legal guardian of all of the enrolled children.

On the basis of the guidelines of the European Society for Pediatric Infectious Diseases [5], severe CAP was defined as the presence of one of the following findings: 1) respiratory distress defined on the basis of age-adjusted tachypnea, peripheral oxygen saturation <93%, cyanosis, retractions, grunting or nasal flaring; 2) a capillary refill time of >2 seconds or dehydration; and 3) vomiting/not feeding.

Upon admission, chest radiographs were obtained in the two standard projections. Eleven pulmonary fields were evaluated by the same blind expert radiologist (MAP): upper right lobe, upper left lobe, right middle lobe, lingula, right hilum, left hilum, right paracardiac, left paracardiac, retrocardiac, right lower lobe, left lower lobe. The presence of atelectasis, pleural effusion and interstitial lung disease was also considered. The distribution of abnormalities was categorised as focal when only one single pulmonary location was involved, and multifocal (unilateral or bilateral) when two or more areas were lesioned.

The children with severe or mild/moderate CAP were compared in order to identify any correlations between CAP severity and the radiological findings. Categorical variables are given as numbers and percentages and were analysed using contingency table analysis with the chi-squared or Fisher’s test, as appropriate. The multivariate odds ratios (OR) of severe CAP, and the corresponding 95% confidence intervals (CIs), were derived using unconditional multiple logistic regression models including terms for atelectasis and pleural effusion. All of the tests were two-sided and a p-value of less than 0.05 was considered statistically significant. The data were analysed using SAS version 9.1 (SAS Institute, Cary, NC, USA) statistical software.

## Results

Seventy-six of the 335 enrolled children (22.7%) fulfilled the criteria for severe CAP. They all had oxygen saturation of <90% and other signs of respiratory distress, including age-adjusted tachypnea, cyanosis, retractions, grunting or nasal flaring; 39 (51.3%) had difficulties in feeding; and 18 (23.7%) were affected by vomiting. None of them had a capillary refill time of >2 seconds or dehydration.

The most frequent radiological presentation was focally distributed parenchymal densities (212, 63.3%), whereas 123 patients (36.7%) showed multifocal consolidations, predominantly bilaterally (85/123, 69.1%). Atelectasis and pleural effusions were detected in respectively 30 (8.9%) and 33 patients (9.8%), and only five radiographs (1.5%) showed interstitial changes. Parenchymal densities were more prevalent in the right than the left lung (263 vs 179), and consolidations were more frequent in the middle lung than in the lower and upper areas (247 vs 176 and 19). The most frequently affected locations were the right lower lobe (75, 22.4%), the right paracardiac field (65, 19.4%), the left lower lobe (63, 18.8%), and the right hilum (61, 18.2%).

Table 1 summarises the associations between CAP severity and the radiological findings. In comparison with the children with mild/moderate CAP, there was a significantly greater frequency of a bilateral multifocal distribution (p = 0.01), the simultaneous involvement of ≥3 sites (p = 0.007), and the involvement of the right hilum (p = 0.02) in those with severe CAP. These findings were confirmed in the multiple logistic regression model, in which severe CAP associated with a bilateral multifocal distribution (OR 2.30; 95% CI, 1.29-4.10), the simultaneous involvement of ≥3 sites (OR 3.01; 95% CI, 1.31-6.94), and involvement of the right hilum (OR 3.25; 95% CI, 1.23-8.54). No association was found between CAP severity and the presence of atelectasis, middle right lobe involvement or pleural effusion.

**Table 1:** Association between CAP severity and radiological findings

**Distribution (n)**	**Severe CAP (%)**	**Mild to moderate CAP (%)**	*P *value*	**Adjusted OR° (95% CI)**
*All locations (n = 335)*	*n = 76*	*n = 259*		
Focal (n = 212)	38 (50.0)	174 (67.2)	0.01	1^§^
Unilateral multifocal (n = 38)	9 (11.8)	29 (11.2)		1.25 (0.53-2.97)
Bilateral multifocal (n = 85)	29 (38.2)	56 (21.6)		2.30 (1.29-4.10)
Focal (n = 212)	38 (50.0)	174 (67.2)	0.007	1^§^
2 locations (n = 94)	26 (34.2)	68 (26.2)		1.68 (0.93-3.04)
≥3 locations (n = 29)	12 (15.8)	17 (6.6)		3.01 (1.31-6.94)
Pattern				
Atelectasis: no (n = 305)	66 (86.8)	239 (92.3)	0.15	1^§^
Atelectasis: yes (n = 30)	10 (13.2)	20 (7.7)		1.13 (0.48-2.62)
Pleural effusion: no (n = 302)	68 (89.5)	234 (90.3)	0.82	1^§^
Pleural effusion: yes (n = 33)	8 (10.5)	25 (9.7)		1.82 (0.81-4.08)
*Only focal locations (n = 212)*	*N = 38*	*N = 174*		
Right lower lobe: no (n = 164)	31 (81.6)	133 (76.4)	0.49	1^§^
Right lower lobe: yes (n = 48)	7 (18.4)	41 (23.6)		0.55 (0.24-1.28)
Left lower lobe: no (n = 181)	33 (86.8)	148 (85.1)	0.78	1^§^
Left lower lobe: yes (n = 31)	5 (13.2)	26 (14.9)		0.92 (0.33-2.58)
Middle lobe: no (n = 179)	36 (94.7)	143 (82.1)	0.08	1^§^
Middle lobe: yes (n = 33)	2 (5.3)	31 (17.8)		0.17 (0.04-0.84)
Right hilum: no (n = 190)	30 (78.9)	160 (92.0)	0.02	1^§^
Right hilum: yes (n = 22)	8 (21.1)	14 (8.0)		3.25 (1.23-8.54)
Left hilum: no (n = 193)	36 (94.7)	157 (90.2)	0.38	1^§^
Left hilum: yes (n = 19)	2 (5.3)	17 (9.8)		0.50 (0.11-2.26)
Right paracardiac: no (n = 176)	30 (78.9)	146 (83.9)	0.46	1^§^
Right paracardiac: yes (n = 36)	8 (21.1)	28 (16.1)		1.49 (0.61-3.63)
Other locations: no (n = 189)	32 (84.2)	157 (90.2)	0.28	1^§^
Other locations: yes (n = 23)	6 (15.8)	17 (9.8)		1.82 (0.66-5.02)
Upper lung: no (n = 209)	38 (100)	171 (98.3)	1.00	1^§^
Upper lung: yes (n = 3)	0	3 (1.7)		-
Middle lung: no (n = 95)	16 (42.1)	79 (45.4)	0.71	1^§^
Middle lung: yes (n = 117)	22 (57.9)	95 (54.6)		1.09 (0.53-2.24)
Lower lung: no (n = 120)	22 (57.9)	98 (56.3)	0.86	1^§^
Lower lung: yes (n = 92)	16 (42.1)	76 (43.7)		1.01 (0.49-2.08)

CAP: community-acquired pneumonia; CI: confidence interval; OR: odds ratio.

*P values were calculated using two-sided chi-squared or Fisher’s exact test, as appropriate.

°Estimates from multiple logistic regression models, including terms for atelectasis and pleural effusion (when appropriate).

§Reference category.

## Discussion

This study shows that a multifocal bilateral distribution, the simultaneous involvement of at least 3 sites, and right hilar consolidation are associated with severe CAP in otherwise healthy children.

Little has so far been published on the subject. Bay *et al*. studied eight children hospitalised because of severe avian influenza H5N1 pneumonia, and found that chest radiography may play an important prognostic role as the predominant radiological manifestations of the most complicated cases were multifocal bilateral consolidations, mainly located in the lower lobes [8]. A retrospective study of 210 children with viral pneumonia found that diffuse areas of air space consolidations were associated with acute respiratory distress syndrome and required mechanical ventilation [9]. No data are available on the association between severe bacterial CAP and radiographic findings, although there are studies showing that chest radiography did not identify any typical or atypical bacterial etiology regardless of CAP severity [10,11]. However, although multifocal bilateral involvement has been previously identified as a severity criteria, to the best of our knowledge this is first study showing a significant association between severity and right hilar involvement. Furthermore, although the atelectases were small or segmental and there were no massive pleural effusions or empyema, we did not find any correlation between CAP severity and the presence of either. In a previous report, Kin Ken *et al*. demonstrated that the frequency of upper lobe involvement was significantly higher among severe cases defined according to WHO but not British Thoracic Society criteria for patients aged ≥1 year [12]. Although in our study the involvement of the upper areas occurred in a limited number of cases, we did not observe this type of association.

Ferrero *et al*. showed that upper lobe affection and pleural effusion were associated with pneumococcal isolation and with pneumococcal bacteremia, although no association with CAP severity has been performed in their study [13]. Michelow *et al*. observed that children with typical bacterial or mixed bacterial/viral infections had the greatest inflammation and disease severity, although none of the radiographic findings appeared associated with disease severity [14]. The limitations of our study include the lack of etiological data and other laboratory results as well as the fact that the information was only collected from otherwise healthy children with no serious underlying disease. Moreover, the mean age of our study population was quite high and patients were all enrolled among children admitted to hospital and followed by the Respiratory Disease Section of the Pediatric Clinic. This means that future research in this area is needed in order to identify possible correlations between the clinical criteria of severity, radiological characteristics, etiological findings and new laboratory biomarkers in order to develop a specific algorithm that can be easily used in clinical practice.

## Conclusions

This study shows that radiological findings such as a multifocal bilateral distribution, the simultaneous involvement of at least three sites and right hilar consolidation are associated with severe CAP in otherwise healthy children, and could be considered markers of disease severity in children with CAP.

## Abbreviations

CAP: Community-acquired pneumonia; CI: Confidence interval; OR: Odds ratio; SD: Standard deviation.

## Competing interests

The authors declare that they have no competing interests.

## Authors’ contributions

MFP drafted the manuscript and enrolled the patients; BL and ML participated in the enrolment of the CAP patients; CG made the statistical analysis; MAP performed and evaluated all the chest radiographs; SE coordinated and supervised the project, co-wrote the draft manuscript, and was head of the pediatric clinic at which the CAP patients were enrolled. All of the authors read and approved the final version of the manuscript.
